# Influence of Education, Cognition, and Physical Disability on Quality of Life of Romanian Patients with Multiple Sclerosis—A Cohort Study

**DOI:** 10.3390/medicina60030386

**Published:** 2024-02-25

**Authors:** Alin Ciubotaru, Emilian Bogdan Ignat, Daniel Alexa, Cristina Grosu, Ioana Păvăleanu, Alina Manole, Alexandra Maștaleru, Maria Magdalena Leon, Daniela Viorelia Matei, Doina Azoicăi

**Affiliations:** 1 Department of Neurology, “Grigore T. Popa” University of Medicine and Pharmacy, 700115 Iași, Romania; alinciubotaru94@yahoo.com (A.C.); alexadaniel2004@yahoo.com (D.A.); fcristina_ro@yahoo.com (C.G.); 2 Department of Neurology, Rehabilitation Hospital, 700661 Iași, Romania; 3 Mother and Child Departament, “Grigore T. Popa” University of Medicine and Pharmacy, 700115 Iași, Romania; ioana_pavaleanu@yahoo.com; 4 Department of Preventive Medicine and Interdisciplinarity, “Grigore T. Popa” University of Medicine and Pharmacy, 700115 Iași, Romania; alina.manole@umfiasi.ro (A.M.); doina.azoicai@gmail.com (D.A.); 5 Departament of Medical Specialities I, “Grigore T. Popa” University of Medicine and Pharmacy, 700115 Iași, Romania; alexandra.mastaleru@gmail.com (A.M.); leon_mariamagdalena@yahoo.com (M.M.L.); 6 Faculty of Medical Bioengineering, “Grigore T. Popa” University of Medicine and Pharmacy, 700115 Iași, Romania; dvm2202@yahoo.com

**Keywords:** multiple sclerosis, quality of life, MSQOL-54, 9HPT, walking speed, 25FWT, cognition, SDMT, education, disability evaluation

## Abstract

*Background and Objectives*: objective measures of disability and neurological impairmentare used to search for disease activity and monitor disease evolution in multiple sclerosis (MS). These are sometimes in disagreement with subjective quality-of-life measures. We aimed to establish the relations between the Multiple Sclerosis Quality of Life instrument (MSQOL-54) and objective measures of neurological impairment. *Materials and Methods*: 107 patients with MS were evaluated with the Single Digit Modalities Test (SDMT) for cognition, Nine Holes Peg Test (9HPT) for upper limb function, 25 Feet Walk Test (25FWT) for gait, and EDSS for global disability in a cohort study. Age and education level were recorded as sociodemographic factors. *Results*: the median EDSS was 3.5 (IQR 2.5); average SDMT score was 30.38 ± 13.54; and 9HPT speed was significantly higher for the dominant upper limb (0.3 ± 0.1 pegs/s versus 0.28 ± 0.11 pegs/s) (*p* = 0.016). The mental health composite score of the MSQOL-54 correlated with the SDMT, education level, and EDSS. Education level correlated with cognition and quality of life. The physical health composite score of the MSQOL-54 correlated with motor-function parameters and with SDMT. The motor-function parameters correlated well among themselves. A linear regression model found an important influence of SDMT and education level on the mental health composite score of the MSQOL-54. Although the linear regression model predicting the physical health composite score from physical disability parameters was statistically sound, none of the determinants had a significant individual influence. *Conclusions*: the subscores of the MSQOL-54 correlated well with the objective parameters. The strongest correlations were those with the cognitive function. Correlations with physical disability were less powerful, probably reflecting their indirect and more limited influence on quality of life compared to cognition and perception of disability.

## 1. Introduction

Multiple sclerosis (MS) is a central nervous system disease characterized by both inflammation and neurodegeneration/axonal loss [1]. It is one of the most important causes of neurological disability in young adults [2], with a higher prevalence in regions with a colder climate (Europe and North America). An estimated 2.3 million patients are suffering from MS worldwide [3]. In Romania, current data estimate prevalence at around 26.1/100,000 [4], and incidence at approximately 0.32/100,000 [4,5].

The impact of MS on quality of life is complex and goes beyond the levels expected from a chronic disease. MS patients have a lower quality of life when compared to persons who suffer from other chronic diseases [6].

In the setting of a personalized, patient-centered therapeutical approach, evaluation of the perceived quality of life in patients with MS can provide additional information about disease/treatment-related burden, as well as a holistic evaluation of treatment effectiveness (acknowledging that a good quality of life is the ultimate goal of any treatment).

Although therapeutic protocols do not officially acknowledge quality-of-life scales and other such parameters as efficacy measures, there is an increasing interest in this field, as shown by the large number of related publications and by the many instruments validated to measure quality of life in MS patients. The Functional Assessment of Multiple Sclerosis (FAMS), Multiple Sclerosis Impact Scale (MSIS), Hamburg Quality of Life Questionnaire in Multiple Sclerosis (HAQUAMS), Multiple Sclerosis Quality of Life Inventory (MSQOLI), the 27-item Multiple Sclerosis Quality of Life Questionnaire (MS-QLQ27), and the Multiple Sclerosis International Quality of Life (MUSIQOL) are some of the scales available in English that could be used to monitor the evolution of the disease or to customize the medical care plan [7,8,9,10,11,12]. However, to our knowledge, the only one validated for the Romanian language is the Multiple Sclerosis Quality of Life Instrument (MSQOL-54) [13].

A person’s ability to cope with the disease (and hence his/her ability to maintain a good quality of life) is subject to numerous influences, both endogenous and exogenous [14,15,16]. While quality-of-life measures constantly include somatic disability and disease duration, education level and cognition could also have an impact on its physical or mental determinants in MS patients [17,18] and, therefore, be important in terms of disease management and progression. Treatment type and sociodemographic factors (sex, age, and education, among many others) might have a more profound effect than anticipated (in terms of disability accumulation, access to treatment, and inclusion) and ultimately on the quality of life of patients, prompting for a more integrated approach [19]. As opposed to quality-of-life tools, objective measurements of motor performance (as are the 9 Hole Peg Test (9HPT), 25 Foot Walk Test (25FWT), or the Expanded Disability Status Scale (EDSS)) primarily highlight the severity of the disease as reflected by the ability to move in the environment or to use the upper limbs (UL). 

Since EDSS (the universally accepted disability measure in MS) is not always satisfactory in capturing clinical differences (especially in cases with gait impairment), 9HPT and 25FWT were introduced, as more sensitive tools to monitor clinical evolution. In 9HPT, the patient is timed as he introduces nine pegs in the holes in a special board [20]. The 25FWT measures the time a patient needs to walk “as fast as he can in a safe pace” the named distance [20]. Neither one of these tests has predefined “pathological” thresholds, as they are mainly used to monitor evolution. SDMT was introduced in order to compensate for another weakness of EDSS—its lack of sensitivity in terms of cognitive dysfunction. Subjects are required to “translate” a string of abstract symbols into digits according to a key [21], with the number of correct replacements constituting the final score. MSQOL-54 is a disease-specific instrument that was developed from the general 36-item Short Form Health Survey to specifically address quality of life aspects in MS [22].

The objective of this study was to evaluate the correlation between quality of life (MSQOL-54 questionnaire) and objective measures of disability in MS subjects—be they physical (9HPT, 25FWT, EDSS), cognitive (Symbol Digit Modalities Test—SDMT), or social (education). We aim to provide insight into the quality of life of Romanian MS patients, as there are no clear, comprehensive data covering this area published yet. Furthermore, we aim to clarify some of the questions related to the ability of MSQOL-54 to correctly reflect changes in various clinical, psychocognitive, or social aspects of life, as existing data is frequently unclear, despite it being a test designed especially for MS. 

## 2. Materials and Methods

This observational cohort study included 107 patients with relapsing-remitting (102 patients, 95.33%) and secondary progressive MS (5 patients, 4.67%) treated in the Department of Neurology, Clinical Rehabilitation Hospital of Romania, Iasi. The study was approved by the Ethics Committee of the Clinical Rehabilitation Hospital Iasi.

To be eligible, the patients had to be more than 18 years old and fulfill the McDonald 2017 criteria for the diagnosis of MS. The exclusion criteria were the patient’s refusal to participate in the study, inability to discern and consent to participate, and any other concomitant condition that could affect the quality of life, including (but not limited to) cardiac, pulmonary, renal disease, or mental health conditions. Subjects were included in order of arrival at the center for their periodical evaluation (all MS patients treated in the service are required to come periodically for clinical tests and evaluation). All subjects were neurologically stable and free of relapse for at least 90 days (3 of the patients (2.8%) had a relapse 6 to 12 months before the evaluation and 104 patients had no documented relapse in the 12 months preceding it). All study participants signed the informed consent.

For the cognitive evaluation, we used the oral form of SDMT (taking into account the number of correctly identified symbols).

Physical disability was quantified using the EDSS score (globally), the 9HPT (measuring the function of the upper limbs), and the 25FWT (for lower limbs function). For the 9HPT, both the dominant and nondominant upper limbs were tested twice, and the average values were used for the statistical analysis. In order to assign a discrete value for the situation of a patient not being able to finalize the test, we have replaced time of completion with speed of completion (measured in pegs per second and calculated as time divided by nine for patients who completed at least one time the test). A value of zero was assigned when patients were not able to finalize the 9HPT at least once.

Similarly, we have used the averaged value of the two 25FWT trials to calculate the average speed (feet/second), assigning a speed of 0 feet/second to patients who were not able to complete the test at least once. Both tests are part of the MSFC (Multiple Sclerosis Functional Composite) and were performed according to the approved guidelines [20]. 

To evaluate the impact of MS on patient’s autonomy and quality of life, we have used the Romanian version of the MSQOL-54 questionnaire [13]. The physical health composite (MSQOL Ph) and the mental health composite (MSQOL M) scores were calculated [22].

Education level (EL) was collected from patient files as years of education and recoded using the major learning cycles in Romania (less than 8 years, 8 years, 12 years, and more than 15 years). Codes were allocated according to the highest graduated cycle (for example 8, 9, 10, or 11 years of school were coded as 8 years, while 12, 13, and 14 as 12—high school).

Treatment was coded in terms of platform treatment (Beta interferons, Glatiramer acetate, Teriflunomide, and Dimethilfumarate) versus high-efficacy treatment (Natalizumab, Ocrelizumab, Cladribine, Fingolimod, and Alemtuzumab). 

Data were statistically processed using IBM’s Statistical Package for the Social Sciences (SPSS) version 23.0 (Copyright IBM Corporation and its licensors 1989, 2019. SPSS and IBM are trademarks or registered trademarks of International Business Machines Corporation, registered in many jurisdictions worldwide). Average values are presented as average ± standard deviation.

The normal distribution of data was tested using the Kolmogorov–Smirnov test. Correlations were assessed using Pearson’s r or Spearman’s rho (the latest being used when at least one of the tested parameters did not have a normal distribution). Correlations were considered to be strong if the correlation coefficient was above 0.5 (or below −0.5 for inverse correlations), moderate for a correlation coefficient between 0.3 and 0.5, and weak for a correlation coefficient below 0.3. Wilcoxon Signed Ranks Test was used to test for significant differences in non-normally distributed samples. Multivariable linear regression was used to model a predictive relationship between MSQOL-54 composite scores and the motor, cognitive, and social determinants.

## 3. Results

The general demographic data of the 107 enrolled patients were sex (68 women and 39 men) and age (41.42 ± 11.22 years).

The first part of the analysis studied any existing relations between the MSQOL M score and its possible predictors/correlators. SDMT score, education level, age, and treatment type were analyzed. All of these parameters, except for education level and EDSS, were normally distributed. 

EL was “less than 8 years” for 3 patients (2.8%), “elementary school” (8 years) for 21 patients (19.63%), “high school” (12 years) for 41 patients (38.32%), and “college or above” (more than 15 years) for 42 patients (39.25%). The treatment type was “platform therapy” for 51 subjects (47.66%) and “high efficacy therapy” for 56 subjects (52.33%). EDSS was between 1 and 7.5 (median 3.5, IQR 2.5), MSQOL M ranged from 10.8 to 115.29 (59.45 ± 24.14), and SDMT scores ranged from 4 to 65 (30.38 ± 13.54). 

**Figure 1:**
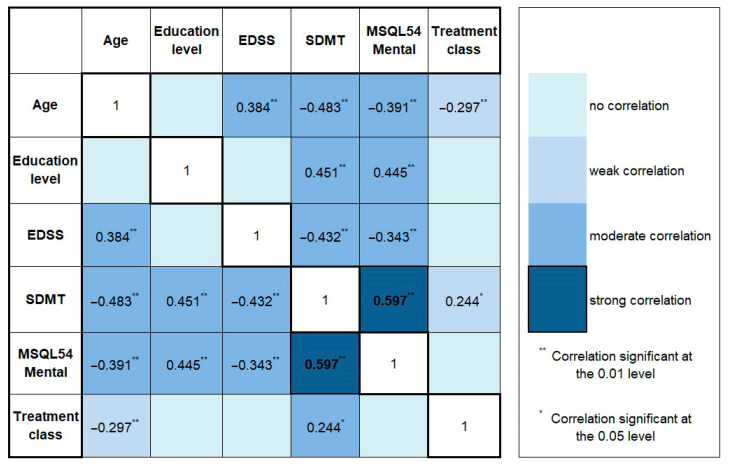
Correlations between MSQOL M, clinical, and cognitive parameters. Abbreviations: MSQL54 Mental—Mental Health Composite Score of the Multiple Sclerosis Quality of Life —54 Instrument; SDMT—Single Digit Modalities Test; EDSS—Expanded Disability Status Scale. Correlation coefficients for strong correlations appear in bold.

The results showed that MSQOL M strongly correlates with SDMT and moderately with EL. Moderately strong inverse correlations were found between MSQOL M and EDSS, MSQOL M, and age, whereas EL moderately correlates with SDMT. EDSS positively correlates with age and negatively with SDMT. SDMT negatively correlates with age. Treatment class inversely correlates with age (high efficacy treatment in younger patients) and correlates with SDMT. All correlation coefficients (Pearson’s r or Spearman’s rho) can be found in Figure 1.

A linear regression model with MSQOL M as the dependent variable and all the other parameters as variables (SDMT, EL, EDSS, age, and treatment class) generated an R = 0.646 (*p* < 0.001) and an adjusted coefficient of determination (R square) of 0.389 with a significance < 0.001. Multicollinearity was excluded (variance inflation factor between one and two for all constants). However, only SDMT and EL had significantly impacted MSQOL M, with SDMT having the largest contribution (see Table 1 for coefficients and significance). Similarly, the automatic linear modeling function in SPSS version 23.0 (using automated data preparation to trim outliers) returned a predictor importance of 0.61 for SDMT and 0.28 for EL, with age, EDSS, and treatment class below the significance level.

The second part of the analysis was centered on the MSQOL Ph score and its possible determinants. EDSS, age, 9HPT speed for the dominant and nondominant upper limb, impossibility to perform the 9HPT, 25FWT speed, and impossibility to perform the 25FWT were analyzed in terms of possible correlations. 

Since most of the test times/average values/speeds from the 9HPT and the 25FWT do not have a normal distribution, the Wilcoxon Signed Ranks test was used. We compared results from the first and the second tests (for both 9HPT and 25FWT), and average times from the dominant and the nondominant upper limbs in 9HPT (Table 2). For both upper limbs, the 9HPT times were significantly smaller in the second trial compared to the first one. There was a significant difference between the average times for the dominant and the nondominant upper limbs. The 9HPT speed was significantly higher for the dominant upper limb. The two trials of the 25FWT were not significantly different. 

**Figure 2:**
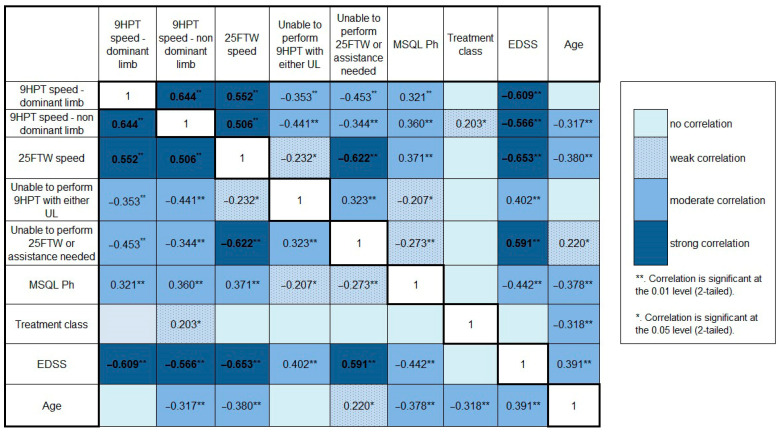
Correlations between MSQOL Ph and physical parameters (Spearman’s rho and significance level) Abbreviations: 9HPT—9 Hole Peg Test; 25FTW—25 Feet Walk Test; MSQL Ph—Physical Health Composite Score of the Multiple Sclerosis Quality of Life —54 Instrument. Correlation coefficients appear in bold in the case of strong correlations.

Correlations between motor tests and scores (9HPT both limbs, 25FWT, and EDSS) were the strongest, with rho values above 0.5. MSQOL Ph scores positively correlated with 9HPT (both upper limbs) and 25FWT and negatively correlated with age (with similar values of the rho coefficient around 0.3 or −0.3, respectively); the inverse correlation with EDSS was the strongest (rho = −0.442). The inability to perform the 9HPT with either upper limb and the need for walk aids/inability to perform the 25FWT showed weak correlations with MSQOL Ph but correlated better with EDSS (especially 25FWT impossibility/need for assistance—rho = 0.591, *p* < 0.001). An unexpected inverse correlation was found between age and 25FWT impossibility/need for assistance, with older patients being less likely to have gait impairment (rho = −0.378, *p* < 0.001). Treatment class did not show significant correlations apart from age, which showed a tendency for younger patients to have higher efficacy treatment in our group. Age inversely correlated with speed for the 9HPT (but the correlation was significant only for the nondominant upper limb) and positively correlated with EDSS. All rho values can be found in Figure 2.

As we did in the case of the relation between MSQOL M and cognitive parameters, a linear regression model was tested with MSQOL Ph as a dependent variable and 9HPT speeds (dominant and nondominant upper limbs), inability to perform 9HPT with either upper limb, 25FWT speed, need for assistance or inability to perform 25FWT, age, and EDSS as predictors. While R = 0.488 (*p* = 0.001), the adjusted coefficient of determination (R square) was 0.239, and none of the determinants had a significant impact on MSQOL Ph except age (standardized beta coefficient −0.23, *p* = 0.028). 

MSQOL M and MSQOL Ph were highly correlated (Spearman’s rho = 0.808, *p* < 0.001). There was a good correlation between SDMT and MSQOL Ph (Spearman’s rho = 0.473, *p* < 0.001), and EL also correlates to MSQOL Ph (rho = 0.327, *p* = 0.001). 

Linear regression with all parameters (both “physical” and “cognitive”—age, education level, 9HPT speed, 25FWT speed, and EDSS) as predictors and MSQOL Ph as the dependent variable generated an R = 0.583 (R^2^ = 0.340, adjusted R^2^ = 0.293), which is statistically significant (*p* < 0.001). Of all the predictors, only SDMT had a significant impact on MSQOL 54 (Table 3).

SDMT strongly correlates with 9HPT speed (both dominant UL—rho = 0.551, *p* < 0.001—and nondominant UL—rho = 0.527, *p* < 0.001) and with 25FWT gait speed (rho = 0.39, *p* < 0.001).

## 4. Discussion

Quality of life is determined by a multifactorial array of elements that combine to influence various aspects of functioning. However, identifying and quantifying the impact of a specific factor is not always easy, as disease understanding changes constantly as are the relations among individual determinants. On the other side, patient’s perceptions are not always in tone with “academic” dogma. We attempted to clarify how various objective parameters (both in terms of mental and physical disability) explain the changes of a patient-centered measure (MSQOL54 questionnaire) in a sample of patients with MS. The MSFC (Multiple Sclerosis Functional Composite) score aims to cover aspects of neurological impairment that the EDSS does not capture in a satisfactory manner. As opposed to 9HPT and 25FWT, the PASAT score (Paced Audio Serial Addition Test), also part of the MSFC, is less widely accepted and used, with SDMT becoming the preferred screening test for cognition in MS [21]. 

In our study, the mental health composite score of the MSQOL54 best correlated with the SDMT (R = 0.597). Correlation with education level was close to that in terms of power (R = 0.445), while its relations with age and EDSS were less tight. SDMT and age also seem to be the most important determinants in the regression model, explaining 39% and 23% (respectively) of the variance of the mental health component of the SDMT. Somehow, surprisingly, SDMT also had the highest correlation score with the physical component of the MSFC (rho = 0.473), more than tests/scores directly related to physical abilities (upper limb function, gait, and EDSS scale). 

As in other pathologies, we expect an impaired upper limb function (especially of the dominant upper limb) to have a significant impact on quality of life, as most of the current basic activities require at least some participation from the upper limbs [23]. Upper limb function was evaluated in our study in terms of dexterity (“skillful and controlled manipulation of a tool or an object by the fingers” [24]) with the 9 Hole Peg Test. When compared to the normative data of the 9HPT, measured times of performance in our group were significantly lower (Wilcoxon Signed Ranks Test *p* < 0.001 for both UL). Normal values and then differences were calculated according to age and sex groups [25]. Pooled average time for the dominant upper limb was 30.31 ± 8.61 s compared to 17.34 ± 0.98 s (an average difference of 12.93 ± 8.5 s) and for the nondominant upper limb, 31.55 ± 9.12 s compared to 18.39 ± 0.98 (average difference of 13.12 ± 8.79 s). Although in MS the main purpose of SDMT is to highlight change over time, our study does not include seriate evaluations that could reveal an eventual progression of the disease. However, the wide distance between expected and measured times of completion (on average, these were at least 70% higher than expected, with virtually all of the patients above the normal range) is suggestive of markedly impaired dexterity in all of the patients for both upper limbs. It is somehow unexpected to find that the 9HPT speed correlates only moderately with quality of life (MSQOL54 physical composite score) and does not have a significant impact on the motor component of MSQOL54 in a regression analysis. Even though median EDSS was 3.5 in our group (below the threshold where gait becomes the exclusive determinant), the strong correlation of 9HPT speed with EDSS is more likely due to the presence of widespread motor impairment in some of the patients (supported by the strong correlation between 9HPT and 25FWT speeds) than to isolated dysfunction of the upper limbs. EDSS (as an indicator of global disability) correlated better with the physical component of MSQOL54 than individual upper and lower limb tests.

The very inhomogeneous performance time of completion of 9HPT in our group (expressed as raw values ranging between 17.31 and 60.24 s in the dominant UL and 18.16 and 63.01 s in the nondominant UL, as well as large values of calculated standard deviations), could also impact the relevance of the statistical analysis.

The 25FWT time in our study was similar to data from much larger groups of MS patients. In [26], the mean baseline score measured for the 25FWT in 2465 patients was 9.2 s, median = 6.1 (standard deviation = 11.0, interquartile range (IQR) = 4.2), while in our group, the mean 25FWT time was 8.58 s (median of 6.71, standard deviation of 5.72, IQR = 4.15). The average speed was 3.66 ± 1.5 feet/s (ambulatory patients only) [26]. In healthy control volunteers, the time of 25FWT ranged from 2.8 to 5.2 s (median = 3.7 s), with an average speed of 6.89 ± 0.98 feet/s [27]. Since impaired gait is potentially one of the most difficult aspects of MS in terms of coping [28], we would expect abnormal 25FWT to correlate with a marked impact on quality of life. In an attempt to establish clinically meaningful benchmarks in MS [29], a 25FWT performance time above 6 s was associated with professional changes due to MS, requiring “some help” with IADL and loss of functional community ambulation, while a time of more than 8 s was associated with permanent disability (collecting Supplemental Security Income), inability to perform IADL, and use of a walker [29]. The number of patients with 25FWT time < 6 s in our study was 35 (32.4%), with 46 patients (42.59%) requiring more than 8 s or being unable to complete the test (reflecting a severe loss of function). When comparing MSQOL Ph in the four groups defined by the time of completion of 25FWT (less than 6 s, 6–8 s, more than 8 s, and unable), average scores decrease in an inverse relation with time. However, scores between neighboring groups were significantly different only when grouped as “<8 s” versus “>8 s or unable” (66.19 ± 21.26 s versus 42.51 ± 22.11 s, *p* < 0.001).

The moderate correlation (rho = 0.371) between the physical component of MSQOL54 and speed (25FWT), as well as the lack of significant influence of gait speed in the regression model, may seem surprising in this context. A possible explanation is that since MSQOL54 is a multidimensional measure, it is expected for the weight of a particular clinical element to dilute. Only 10 questions carrying a maximum penalty of 17 points (out of 100) from the Physical Health Composite Score directly relate to gait/lower limb function. Contribution to other items is certain, but in an indirect manner, that combines the influence of gait capacity with other subjective or objective parameters, thus attenuating its abnormalities. In opposition, there are only four questions directly linked to cognition (with a maximum of 15 points in the Mental Health Composite Score), but they are not taken into account for the Physical Composite. The relatively stronger correlation between SDMT and the Physical Composite Score (as compared to physical measures) can be related on one side to a more tight relation between subjective perception of disability and the MSQOL54, but also to the way brain resources are used during a specific task. Parallel activities compete, and simultaneous performance of more than one task is often associated with worse performance For example, walking on a treadmill while counting backward led to reduced speed, while walking over normal ground decreased cognitive performance in comparison with walking on a treadmill (which is less demanding in terms of attention) [30]. Different types of cognitive activities have different consequences over gait, as does age [31]. Decision-making impairment in older adults led to poorer performance during precision stepping and reaction-time tasks [32]. Although the separate performance of motor and cognitive tests during our study does not allow a direct inference on motor–cognitive interference, we found a stronger correlation between SDMT and upper limb motor tests compared to the correlation with gait speed. In comparison with upper limb activities that require much more precise regulation, control of gait relies more on rhythmic than on discrete movement and probably has a more automated control [33], being potentially less dependent on central systems integrity [34]. This bidirectional relation of interdependence between motor and cognitive impairment in terms of daily performance may explain part of the complicated relationship between basic motor measures, cognition, and quality of life. 

Not having included measures of fatigue and emotional alteration (and most of all depression) is a weakness of our study, as they can change both the actual and perceived level of disability. Fatigue is an important issue in MS and may interfere with many elements taken into account in quality-of-life assessments [35]. Fatigue and fatigability can be hidden determinants of perceived physical, emotional, and cognitive disability, and tests that are quick to perform (as those that we have used) may not capture this interaction (in our study the performance times decreased in the second trial of both 9HPT and 25FWT, ruling out fatigability). 

In one study that assessed the contribution of MS clusters of symptoms on its consequences [36], quality of life was best correlated with perceived health, then with illness intrusiveness, walking capacity, and fatigue. While functional walking capacity was affected mostly by the “motor cluster” (including spasticity and balance problems), quality of life was better predicted by the “physical” cluster (pain, fatigue, and sleep disorders) and the “cognitive/emotional” cluster (depression, anxiety, cognitive impairment, and irritability) [36]. Similarly, in another study, quality of life was more influenced (as compared to physical disability measures, and notably EDSS) by factors such as number of complaints, activities of daily living, depression, fatigue, family status, physical activity, and occupation [37,38]. In [39], quality of life (evaluated by the MusiQL score) failed to mirror changes in EDSS, while in another study [40], EDSS only correlated with the physical health composite, but not with mental health. In our subjects, EDSS correlated with both the physical and mental subscores of the MSQOL54, with a slightly stronger relation with the physical component. 

The standard tests that we used in this study have their limitations. For instance, the 9HPT may not fully capture the full spectrum of manual dexterity of the upper limb function, as it focuses primarily on manipulation tasks. The SDMT, designed to measure cognitive processing speed, might not adequately assess other cognitive domains, such as memory or executive function, and could be sensitive to cultural and educational factors, limiting its comprehensive evaluation of cognitive abilities. Similarly, the 25FWT may lack sensitivity to subtle changes in lower motor force or coordination and may not fully represent real-world ambulatory capabilities, as it is an indoor test. It is essential to be aware that these limitations could be related at least in part to some of the incongruencies between quality-of-life measures and precise/narrow objective parameters. 

Our study has limitations that impose caution in generalizing our conclusions. Certain particular aspects of the subject group do not replicate the situation in the general population. As such are the proportions of highly educated subjects as well as the proportion treated with high efficacy therapy. The proportion of more severely impaired patients in our group may also be lower than that in the general MS patient population, due to their decreased ability to come to the site (and to be included in the study). Most of all, the low number of subjects allows for only a prudent tentative to generalize our findings. The results lead us to conclude that, while there is a clear correlation between physical and cognitive objective measures and quality of life, this relation appears far less tight than expected. The inadequacy of the measures we have chosen to evaluate is one possible explanation. It is more acceptable that 9HPT and 25FWT mirror inaccurately the motor deficit in the upper and lower limbs compared to it having only a minor influence on quality of life. Another possibility is that the parameters MSQOL54 uses as surrogates to quality of life are mostly compound entities that result from multiple interactions and, as such, respond less to changes in individual basic parameters. With the exception of SDMT (which can be used to accurately predict “mental” aspects of quality of life), we were not able to establish the precise relation between “physical” aspects of quality of life and measures of physical disability.

## 5. Conclusions

Education level and SDMT correlate well with the mental health composite score of the MSQOL54 and can predict its values in a linear regression model. EDSS, 9HPT speed, and 25FWT speed, as well as SDMT, correlate with the physical health composite score of the MSQOL54. We found a more clear relation between quality of life and cognition compared to that with physical disability. 

Since current routine clinical measures are efficacy measures and are surrogates for treatment goals, and since improving the quality of life should be the ultimate goal of a noncurative treatment, further studies are required to clarify whether these can be used to infer future quality of life or whether better measures should be developed. Establishing a clear relationship between medical parameters and a measure of quality of life is essential to validate such a measure. Quality of life and the way it changes in relation to various interventions is a central point in designing health policies. Beyond immediate treatment decisions and protocols, the quality of life in MS is subject to wider political and economic influences, and providing appropriate information is essential when attempting to change health policies.

## Figures and Tables

**Table 1:** Coefficients for linear regression (MSQOL M in relation to age, EDSS, EL, and treatment class, SDMT). Abbreviations: MSQOL M—Mental Health Composite Score of the Multiple Sclerosis Quality of Life -54 Instrument; SDMT—Single Digit Modalities Test; EDSS—Expanded Disability Status Scale.

	Unstandardized Coefficients		Standardized Coefficients	t	Sig.	95.0% Confidence Interval for B	
	B	Std. Error	Beta			Lower Bound	Upper Bound
MSQOL M (Constant)	32.974	14.098		2.339	0.021	5.007	60.941
SDMT	0.701	0.179	0.393	3.905	0.000	0.345	1.057
Treatment class	0.037	3.915	0.001	0.009	0.993	−7.730	7.803
Education level	6.755	2.498	0.232	2.704	0.008	1.800	11.710
EDSS	−1.145	1.227	−0.081	−0.933	0.353	−3.580	1.290
Age	−0.284	0.198	−0.132	−1.436	0.154	−0.677	0.108

**Table 2:** Results from both trials of the 9HPT test (time and speed) and the 25FWT (time) and the significance of comparisons (Wilcoxon Signed Ranks test, two-tailed *p*). Abbreviations: 9HPT—9 Hole Peg Test; 25FWT—25Feet Walk Test; SD—standard deviation; IQR—interquartile range.

9HPT				
Dominant upper limb first trial		Dominant upper limb second trial		Significance
Average ± SD (seconds)	Median (IQR)	Average ± SD (seconds)	Median (IQR)	
32.14 ± 9.06	31 (11.96)	28.51 ± 8.78	26.19 (10.54)	*p* < 0.001
Nondominant upper limb first trial		Nondominant upper limb second trial		Significance
Average ± SD (seconds)	Median (IQR)	Average ± SD (seconds)	Median (IQR)	
32.04 ± 9.23	30.6 (12.46)	31.07 ± 9.44	29.3 (11.65)	*p* = 0.004
Dominant upper limb average time		Nondominant upper limb average time		Significance
Average ± SD (seconds)	Median (IQR)	Average ± SD (seconds)	Median (IQR)	
30.13 ± 8.63	29.135 (10.04)	31.54 ± 9.3	31.265 (10.66)	*p* = 0.039
Dominant upper limb average speed		Nondominant upper limb average speed		Significance
Average ± SD (pegs/second)		Average ± SD (pegs/second)		
0.3 ± 0.1		0.28 ± 0.11		*p* = 0.016
25FWT				
First trial		Second trial		Significance
Average ± SD (seconds)	Median (IQR)	Average ± SD (seconds)	Median (IQR)	
7.76 ± 6.65	6.1 (3.75)	7.64 ± 5.7	6.43 (4.37)	*p* = 0.728

**Table 3:** Coefficients for linear regression (MSQOL Ph in relation to age, EDSS, EL, SDMT, 9HPT speed for both limbs, and 25FWT speed). Abbreviations: MSQOL Ph—Physical Health Composite Score of the Multiple Sclerosis Quality of Life -54 Instrument; SDMT—Single Digit Modalities Test; EDSS—Expanded Disability Status Scale; 9HPT—9 Hole Pegs Test; UL—upper limb; 25FWT—25 Feet Walk Test.

	Unstandardized Coefficients		Beta Coeffic.	T	Sig.	95.0% Confidence Interval for B	
	B	Std. Error				Lower Bound	Upper Bound
Dependent: MSQOL Ph	40.618	17.526		2.318	0.023	5.842	75.394
Age	−0.233	0.223	−0.107	−1.044	0.299	−0.675	0.210
Education level	5.501	2.790	0.187	1.971	0.051	−0.036	11.037
SDMT	0.522	0.224	0.289	2.329	0.022	0.077	0.966
EDSS	−2.638	1.776	−0.185	−1.485	0.141	−6.162	0.887
9HPT speed dominant UL	−22.647	29.529	−0.097	−0.767	0.445	−81.239	35.946
9HPT speed nondominant UL	5.119	23.620	0.023	0.217	0.829	−41.747	51.985
25FWT speed	1.308	1.550	0.099	0.844	0.401	−1.766	4.383

## Data Availability

Data can be obtained on request from the corresponding author.
